# The First Stages of Nanomicelle Formation Captured in the Sevoflurane Trimer

**DOI:** 10.1021/acs.jpclett.2c00671

**Published:** 2022-04-21

**Authors:** Amanda L. Steber, Wenqin Li, Brooks H. Pate, Alberto Lesarri, Cristóbal Pérez

**Affiliations:** † Departamento de Química Física y Química Inorgánica, Facultad de Ciencias-I.U. CINQUIMA, Universidad de Valladolid, E-47011 Valladolid, Spain; ‡ Department of Chemistry, University of Virginia, Charlottesville, Virginia 22904-4319, United States

## Abstract

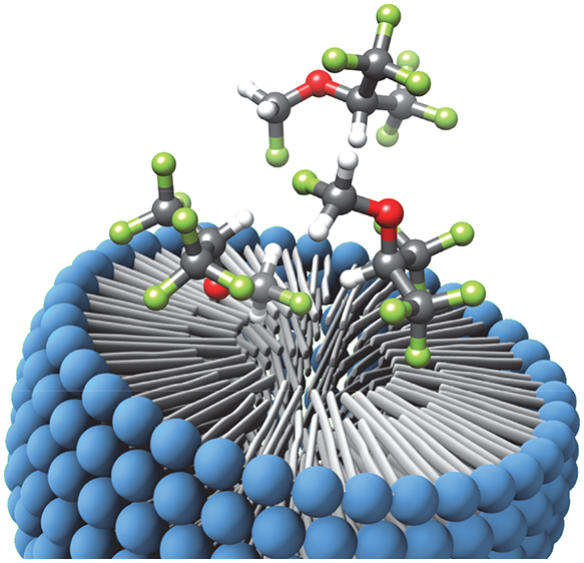

Self-aggregation of sevoflurane, an inhalable, fluorinated anesthetic, provides a challenge for current state-of-the-art high-resolution techniques due to its large mass and the variety of possible hydrogen bonds between monomers. Here we present the observation of sevoflurane trimer by chirped-pulse Fourier transform microwave spectroscopy, identified through the interplay of experimental and computational methods. The trimer (>600 Da), one of the largest molecular aggregates observed through rotational spectroscopy, does not resemble the binding (C–H···O) motif of the already characterized sevoflurane dimer, instead adapting a new binding configuration created predominantly from 17 CH···F hydrogen bonds that resembles a nanomicellar arrangement. The observation of such a heavy aggregate highlights the potential of rotational spectroscopy to study larger biochemical systems in the limit of spectroscopic congestion but also showcases the challenges ahead as the mass of the system increases.

Inhaled anesthetics are broadly used to suppress the activity of the central nervous system, induce loss of consciousness, and facilitate several medical procedures in a painless manner. While the molecular mechanisms of anesthesia are not generally known, several studies have identified a number of specific molecular targets like γ-aminobutyric acid, glutamate, and acetylcholine receptors^1^ as well as voltage-gated ion channels^2^ as binding sites for general anesthesia at a molecular level. The interaction between general anesthetics and ligand/voltage-gated ion channels is a characteristic example of a complex molecular docking mediated by weak non-covalent interactions, where hydrogen bond and weaker dispersive contacts enable molecular recognition.^3^ These prototypical bindings represent local interactions in the active binding sites, and a deeper understanding of the interactions between a particular anesthetic and the target receptor would implicitly lead to a better insight of their biological activity. Of particular interest is the chirality synchronization phenomenon involving achiral building units and how this modulates self-aggregation.^4^ However, observing and modeling these subtle interactions is usually challenging, as they are normally assisted or hindered by water molecules or other binding partners in physiological media. A valuable tool to rationalize these mechanisms is to isolate the molecular partners of interest in the gas phase and use high-resolution techniques for their subsequent observation and characterization. Among these techniques, broadband rotational (CP-FTMW) spectroscopy has been shown to provide insightful information about the structure, binding topologies, and molecular interactions in molecules^5−7^ and molecular aggregates^4,8−14^ of increasing size. The technique’s high sensitivity and high dynamic range allow for observations of these larger systems. Only recently, this technique has also been extended to perform chirality-sensitive measurements by means of three-wave mixing^15,16^ and chiral tagging experiments.^17,18^

Sevoflurane (Figure 1) is a fluorinated ether and one of the most common inhalational anesthetics used for the induction and maintenance of general anesthesia. The molecule was previously studied by rotational spectroscopy to gain information about the monomer’s intrinsic molecular properties.^19^ This study found that, despite its several degrees of freedom, sevoflurane predominantly adopts a single conformation with two equivalent torsional minima (mirror image of each other) separated by a 17.8 kJ/mol potential barrier (see Figure S1). This so-called transient chirality was later captured through the observation of two sevoflurane dimers (homo- and heterochiral), where the two enantiomeric forms were stabilized by non-covalent interactions.^20^ Here we exploit the sensitivity of CP-FTMW spectroscopy to go one step further, and we present the observation of the sevoflurane trimer. This investigation has been performed based on comparisons with high-level quantum-chemistry calculations. We observed that the transient chirality of the monomer is frozen to form a trimer nanomicellar structure where all of the acidic hydrogens point inward in the cluster, while the fluorine atoms form the electron-rich outer layer. This arrangement primarily creates a network of weak CH···F hydrogen bonds involving isopropylic hydrogen bonds that hold together the monomers. They are further anchored by weaker hydrogen bonds involving the aliphatic proton donors in the molecule. Interestingly, the C–H···O based topologies of the previously observed dimers are absent in the structure of the trimer and the oxygen atoms do not play a role in stabilizing the structure. We observed that the monomers slightly distort to form the trimer, but the energy difference that is incurred is compensated for by maximizing interactions between monomers. With rotational constants on the order of 100 MHz and below, the rotational partition function increases considerably, which impacts the overall intensity of the rotational spectrum. The large dynamic range and sensitivity attained in this measurement made it possible to observe this intrinsically weak, low-abundance trimer. This study showcases the size (>600 Da) of molecules that can be studied nowadays with rotational spectroscopy. The current results usher in a new range of feasible molecular target masses for rotational spectroscopy. With these new target systems available, rotational spectroscopy has a newfound potential for analytical applications in the pharmaceutical industry. However, to make a confident, conclusive pairing of the spectrum with a theoretical structure, the attained accuracy of quantum chemistry must be preserved or even improved for ever larger molecules. This study highlights the challenges of molecular identification using rotational spectroscopy paired with theoretical calculations.

**Figure 1:**
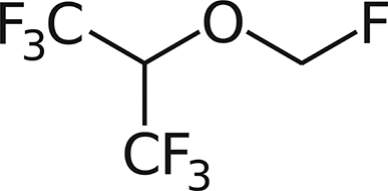
Molecular formula of the volatile anesthetic sevoflurane.

The broadband spectrum shown in Figure 2 was measured by the CP-FTMW spectrometer at the University of Virginia.^21,22^ A mixture of 0.2% sevoflurane vapor (brand name Ultane, Abbott Laboratories, 98+%) in neon was expanded at ca. 6 atm backing pressure, and 9.1 million acquisitions were collected and averaged in the time domain. The spectrum covers the 2–8 GHz frequency range, and it is particularly well-suited for large systems. More experimental details have been previously reported.^20^ This spectrum contains 9600 lines at a signal-to-noise ratio (SNR) of 4:1 or better. Even after removing the monomer, the two dimers, and all of the corresponding isotopologues, >5000 lines remained unassigned. Triggered by the emergence of new methods to sample the potential energy surface (PES) of molecules and molecular complexes and the huge amount of unassigned lines in the spectrum, we performed a computational investigation of the trimer’s PES using the GFN-xTB program with Grimme’s conformer-rotamer ensemble sampling tool CREST.^23^ This search provided 264 initial candidate structures that were further optimized using the DFT hybrid functional B3LYP with Grimme’s dispersion correction D3 and Becke-Johnson damping (B3LYP-D3(BJ)) and the def2-TZVP basis set. Frequency and domain-based local pair-natural orbital coupled cluster perturbative triple-excitations method (DLPNO-CCSD(T)) calculations with the def2-TZVPP basis set and the resolution-of-identity (RIJCOSX) approximation (implemented in ORCA^24,25^) were also performed to examine the stability of the predicted global minimum structure. Binding energies were also calculated accounting for basis set superposition error (BSSE) with the counterpoise approximation.^26^ The results are reported in Table S1. We considered only isomers within a relative energy range of 3 kJ/mol of the lowest energy conformer. Higher energy conformers are not expected to be sufficiently populated in the pulsed molecular expansion.^27^ The relevant parameters for a rotational study are reported, which include rotational constants, electric dipole magnitudes in the principal inertial axes system, and energy order. These sets of predicted rotational parameters were used as initial guesses for plausible identifications in the experimental spectrum.

**Figure 2:**
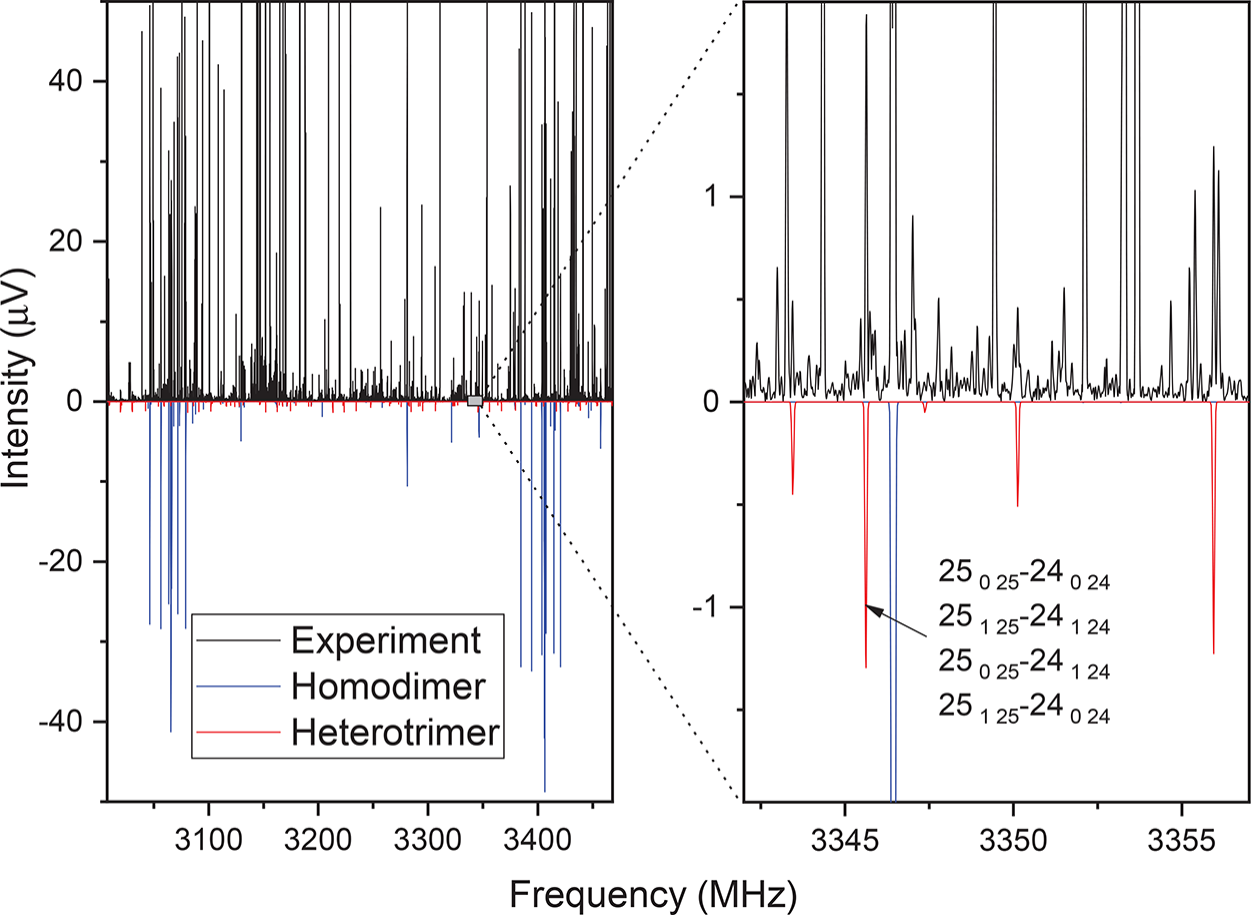
Broadband rotational spectrum of sevoflurane. The gray trace is the experimental spectrum (9.1 million acquisitions). The blue and dark red traces in negative scale correspond to simulations based on experimental parameters for the sevoflurane homodimer and the trimer, respectively. The rotational temperature is fixed to 1.5 K. In the right panel, the complete coalescence of the *a*–*b* asymmetry quartets is shown. The signal-to-noise ratio of the trimer is roughly one order of magnitude lower than that of the dimers.

Given the high line density, computer-assisted^28,29^ searches were performed taking into consideration the predicted selection rules. This procedure allowed us to achieve a successful assignment of the sevoflurane trimer. The initial rotational parameters were subsequently refined in PGOPHER^30^ in an iterative fitting procedure using a semirigid rotor Hamiltonian in the A-reduction (see Tables S2-S3 for differences with predictions and the calculated cartesian coordinates of the trimer). The complete list of transitions is reported in Table S4. To give an idea of the low intensity of the spectrum, the right panel of Figure 2 shows a small portion containing transitions assigned to the trimer. As shown, the observed SNR is roughly 9:1 for the most intense transitions. This is due to both the low abundance of the trimer and the large rotational partition function, ∼(*ABC*)^−1/2^. The latter typically hinders the observation of larger clusters; however, we observed the asymptotic effect of a high-*J* rotor previously described^31^ that simplifies the spectrum and increases the attained sensitivity. This effect produces the gradual coalescence of the asymmetry quartets (see Figure 2, right panel) in two limits, oblate (*J* = *K*_*c*_) and prolate (*J* = *K*_*a*_) with a 2*C* and 2*A* spacing between *J*’s, respectively. Moreover, the observed intensity benefits from this transition coalescence increasing the effective dipole moment following (μ_*a*_^2^ + μ_*b*_^2^)^1/2^ and (μ_*b*_^2^ + μ_*c*_^2^)^1/2^ for the oblate and prolate limits, respectively. In the current spectrum, we observed the oblate limit behavior where the transitions appear spaced by ∼132 MHz, which corresponds to a 2*C* spacing. This effect likely contributed to the detection of the current trimer.

The experimental parameters as well as those obtained from theory are compared in Table 1. The identification of the observed trimer is based on the excellent agreement between the experimental constants and those obtained from quantum-chemistry calculations for isomer II. We observe only small deviations in experiment–theory of 0.24, 1.17, and 0.89% for the *A*, *B*, and *C* rotational constants, respectively. Additionally, we only observed *a*-type rotational transitions. This is in agreement with the predicted magnitude of the dipole moment components. The other transition types were not observed due to the fact that the intensity scales with the square of the dipole moment^21^ and the current SNR. Considering the reported values in Table S1 for this trimer, (μ_*a*_/μ_*b*_)^2^ and (μ_*a*_/μ_*c*_)^2^ are 10 and 7.4, respectively. As shown in Table S1, this system poses a challenge for theoretical methods. Isomer I is essentially isoenergetic with II and the energy order between them varies upon the calculation of choice. Nevertheless, isomer I is not observed due to the lower magnitude of its dipole moment components along with the experimentally observed low line intensity of isomer II. The main challenge here is the reliable, conclusive differentiation between isomers II and IV, as the rotational constants and dipole moment components are very similar and theory predicts them to be less than 1 kJ/mol apart. To test theory’s ability to accurately predict rotational constants with increasing size, we performed a reoptimization of the sevoflurane dimers^20^ at the same level of theory as that of the present study. The results are shown in Table S2. There is a remarkable improvement in the rotational constant relative errors. At the B3LYP-D3(BJ)/def2-TZVP level of theory, the observed errors range from 0.70 to 1.32%. A similar analysis was carried out for both isomers II and IV. While for isomer II all three percentage differences fall into this range, both the *A* and *B* rotational constants for isomer IV exhibit larger errors. Based on this excellent agreement, isomer II can be identified as the carrier of the experimentally observed spectrum, and in what follows, it will be used to discuss the binding topologies of the trimeric nanomicelle. However, this highlights the difficulties when making conclusive assignments as the molecular size increases.

**Table 1:** Experimental Rotational Parameters for the Observed Sevoflurane Trimer Compared to Those from B3LYP-D3(BJ)/def2-TZVP Calculations^a^

	experimental	B3LYP-D3(BJ)/def2-TZVP
*A* (MHz)	115.68934(23)	115.41
*B* (MHz)	91.896543(63)	90.82
*C* (MHz)	66.200589(46)	65.61
Δ_*J*_ (kHz)	0.001636(40)	0.0030
Δ_*JK*_ (kHz)	0.00533(16)	–0.0012
Δ_*K*_ (kHz)	–0.00348(73)	–0.0010
δ_*J*_ (kHz)	0.000361(22)	0.00043
δ_*K*_ (kHz)	0.00227(13)	0.0028
|μ_*a*_| (D)	observed	1.86
|μ_*b*_| (D)		0.62
|μ_*c*_| (D)		0.73
σ (kHz)	6.6	
*N*	360	

a *A*, *B*, and *C* are the rotational constants. Δ_*J*_, Δ_*JK*_, Δ_*K*_, δ_*J*_, and δ_*K*_ are the centrifugal distortion constants. |μ_*a*_|, |μ_*b*_|, and |μ_*c*_| are the magnitudes of the projections of the electric dipole moment onto the principal inertial axes. σ is the rms deviation of the fit, and *N* is the number of transitions in the fit.

In order to help map out the interactions at play, we performed a non-covalent interaction (NCI) analysis^33^ of the electron density topology in the cluster. This analysis identified up to 17 CH···F interactions, ranging from 3.66 to 2.30 Å for the shortest hydrogen bond. Among them, nine contacts are below the sum of the van der Waals radii (2.67 Å), which correspond to the stronger interactions. The results of this analysis are illustrated in Figure 3 where the interactions are represented as colored surfaces ranging from blue to red for attractive and repulsive interactions, respectively. We note that the strongest interactions involve the perfluoro isopropylic hydrogen atoms and the fluoromethoxy groups. These six hydrogen bonds are shown as dashed red lines in Figure 3, and they exhibit distances shorter than 2.45 Å between the atoms. The rest of the 17 interactions are not shown for the sake of clarity. Unlike the previously observed dimers, where the oxygen atoms are one of the main contributors to the stabilization of the structure through a CH···O hydrogen bond in both the hetero- and homodimer, the trimer presents an arrangement that does not involve interactions with the oxygen atoms. Instead, the monomer subunits adopt a micellar-like configuration where all three isopropylic hydrogen atoms point inward and set the overall topology. This is highlighted in Figure 3 with an orange transparent disc that connects the three atoms acting as donors and establishing CH···F contacts with neighboring fluorine atoms. In both monomer one (left foreground) and two (background) in Figure 3, the isopropylic atom bridges the two ends of the adjacent monomer, that is, the CF_3_ and the fluoromethoxy groups, repectively. Interestingly, monomer three (right foreground) links together monomers one and two and interacts with their corresponding fluoromethoxy groups simultaneously (see the rotatable 3D Figure S2) These two interactions are among the shortest contacts in the cluster at 2.41 and 2.33 Å with respect to monomers one and two. These directional, relatively strong interactions compensate for the deformation with respect to the isolated, more stable gas phase form that each monomer undergoes upon complexation.^20^ We observe that, as the ∠COCF dihedral angle, i.e., the relative orientation of the fluoromethoxy group, changes in each monomer, the deformation energy incurred ranges from 0.4 kJ mol^–1^ for monomer one to 2.2 kJ mol^–1^ for monomer three. This particular angle deviates from the most stable conformer by up to 11° for monomer three. Overall, the deformation energy amounts to 4 kJ mol^–1^, which must be compensated for upon cluster formation in order for this trimer to survive. The compensation comes from the hydrogen bond interactions that are established. Indeed, we find that the binding energy is 55.2 kJ mol^–1^ which more than compensates for the small deformations in the monomer units and promotes monomer aggregation.

**Figure 3:**
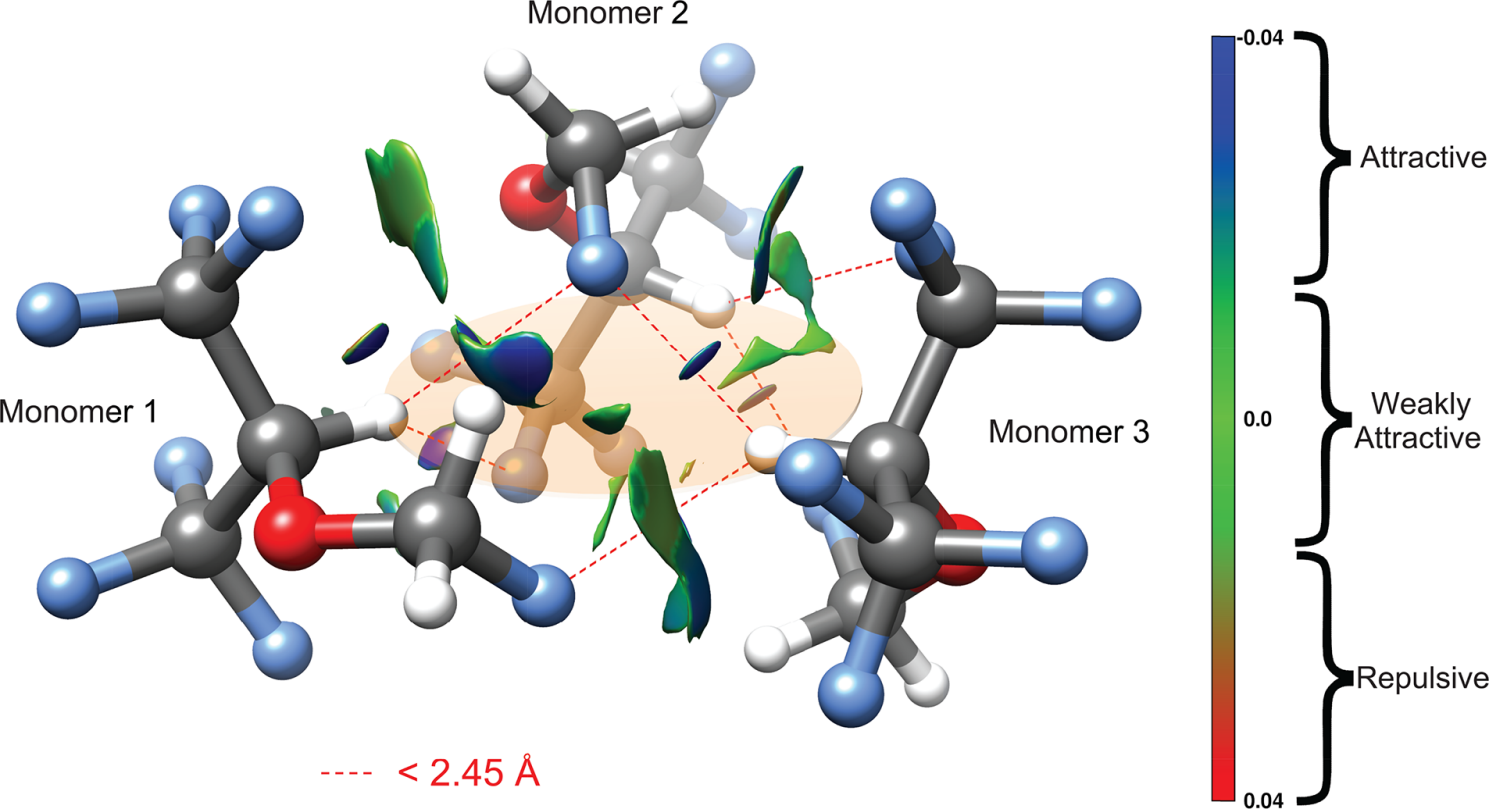
Structure of the observed sevoflurane trimer from B3LYP-D3(BJ)/def2-TZVP calculations. The dotted lines show the interactions below 2.45 Å, and they always involve an isopropylic hydrogen atom. These three hydrogen atoms point to the inner part of the cluster, highlighted by the orange transparent disc for clarity. The NCI interactions are also shown as colored surfaces. These interactions range from attractive CH···F interactions shown in blue to repulsive interactions, of which none are visible, in red.

In summary, we have detected and characterized the trimer of the widely used volatile anesthetic molecule sevoflurane using broadband rotational spectroscopy assisted by novel conformational sampling tools and quantum-chemical calculations. The observed trimer is one of the largest aggregates ever characterized by rotational spectroscopy, and it entails a leap forward in the affordable molecular masses (up to 600 Da) that can nowadays be studied using this technique. The structure of the cluster is stabilized by 17 CH···F interactions, with the more hydrophilic hydrogens pointing toward the center of the cluster and anchoring the three monomers together. Unlike in the previously observed sevoflurane dimers, the trimer does not involve stabilizing interactions with the oxygen atoms, as they are all oriented to the outer part of the structure. The observation of the present trimer emphasizes how the emergence of collective aggregation patterns, generally observed in large micellar structures, may start from a reduced number of molecules, building upon the cooperative use of non-covalent interactions. Our results show the potential of rotational spectroscopy for the analysis of molecules and molecular clusters of increasing sizes but also highlight the challenges for unambiguous identification of molecular structures as the size increases. These species move us closer to understanding more biologically relevant interactions and configurations, and they showcase rotational spectroscopy’s advantageous features for not only fundamental and structural sciences but also analytical applications. The successful combination of sensitive instrumentation with faster and more accurate theoretical tools promises exciting findings in the future.

## Supporting Information Available

Calculated rotational parameters, list of the observed rotational transitions, theoretical Cartesian coordinates of the observed sevoflurane trimer, potential energy scan for the sevoflurane monomer, and rotatable 3D figure of the observed trimer (PDF)
