# Supplementary material for: The First Stages of Nanomicelle Formation Captured in the Sevoflurane Trimer
Source: J Phys Chem Lett. 2022 Apr 21;13(16):3770–5. doi: 10.1021/acs.jpclett.2c00671 (PMC9059180; doi:10.1021/acs.jpclett.2c00671)
Supplement: Supplementary file 1 — jz2c00671_si_001.pdf [file jz2c00671_si_001.pdf]

Supporting information for

The First Stages of Nano-micelle Formation Captured in the Sevoflurane Trimer

Amanda L. Steber[†], Wenqin Li[†] , Brooks H. Pate[‡], Alberto Lesarri[†*] , and Cristóbal Pérez[†*]

[†]Dr. Amanda L. Steber, Wenqin Li, Prof. Dr. Alberto Lesarri, Dr. Cristóbal Pérez, Departamento de Química Física y Química Inorgánica, Facultad de Ciencias-I.U. CINQUIMA, Universidad de Valladolid, E-47011 Valladolid, Spain. E-mail: alberto.lesarri@uva.es, cristobal.perez@uva.es

[‡]Prof. Dr. Brooks H. Pate, Department of Chemistry, University of Virginia, Charlottesville, VA 22904-4319, USA

Figure S1. Potential energy scan for the interconversion of the two transient enantiomers of sevoflurane at B3LYP-D3(BJ)/cc-pVDZ level of theory.

Figure S2. Rotatable 3D pdf with the structure of the observed sevoflurane trimer (isomer II) calculated at the B3LYP-D3(BJ)/def2-TZVP level of theory.

Table S1. Theoretical results at the B3LYP-D3(BJ)/def2-TZVP level of theory. DLPNO-CCSD(T) energy calculations. BSSE complexation energies.

Table S2. Error percentage in the rotational constants for the sevoflurane dimers and trimer.

Table S3. Cartesian coordinates for the observed sevoflurane trimer (isomer II) at the B3LYP-D3(BJ)/def2-TZVP level of theory.

Table S4. Observed rotational transitions and residuals (in MHz) for Sevoflurane trimer II.

Figure S1. Potential energy scan for the interconversion of the two transient enantiomers of sevoflurane at B3LYP-D3(BJ)/cc-pVDZ level of theory.

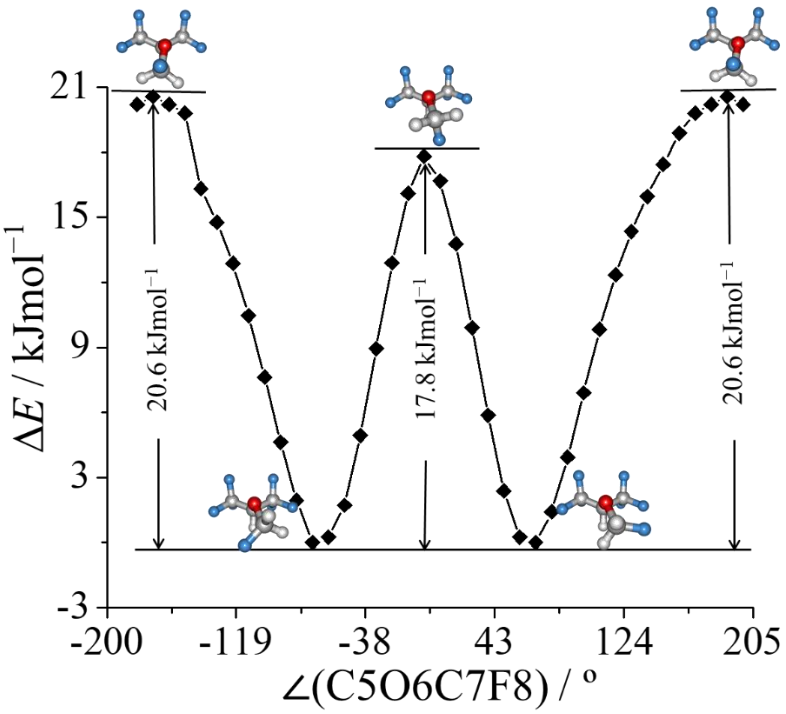

Figure S2. Rotatable 3D pdf with the structure of the observed sevoflurane trimer (isomer II) calculated at the B3LYP-D3(BJ)/def2-TZVP level of theory.

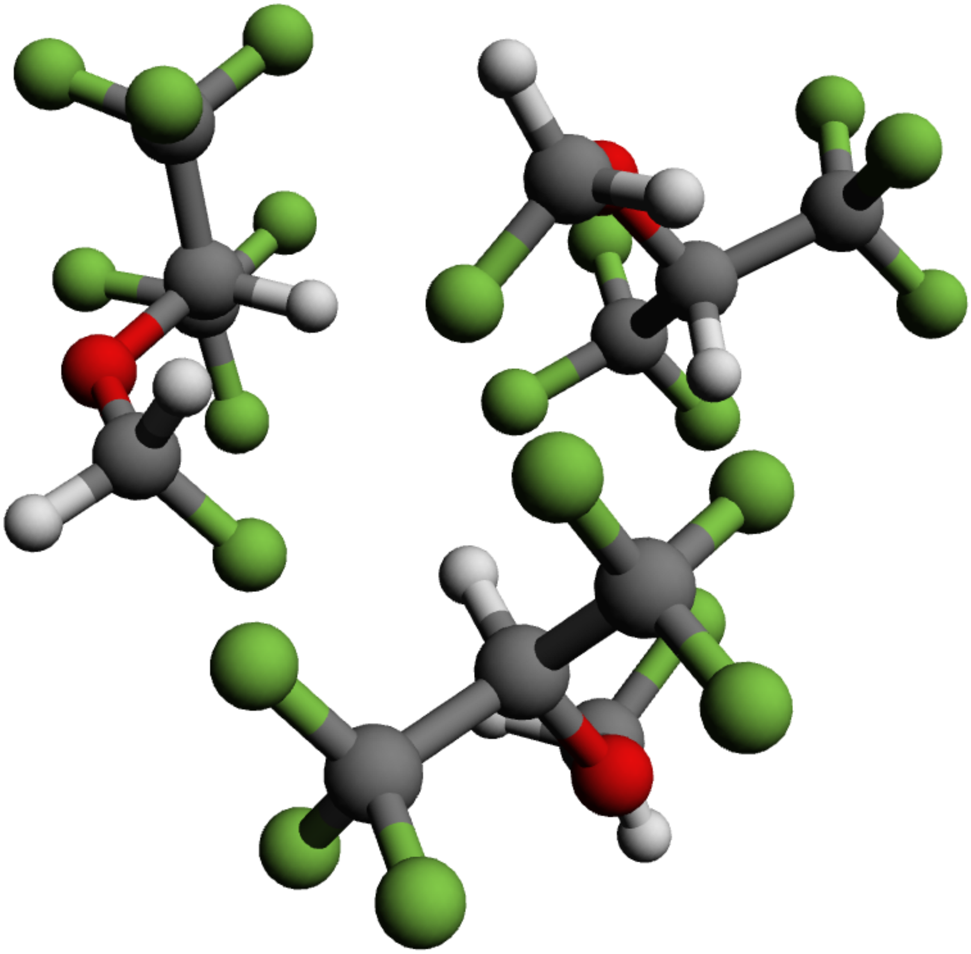

Table S1. Theoretical results at the B3LYP-D3(BJ)/def2-TZVP level of theory. DLPNO-CCSD(T) energy calculations. BSSE complexation energies.

Isomer	I	II	III	IV	V
	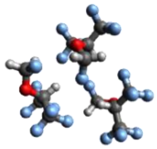	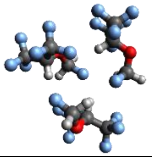	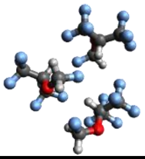	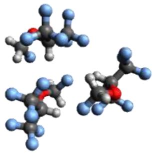	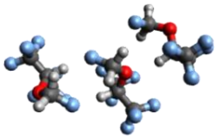
[a]A / MHz	107.1	115.4	108.6	113.0	187.4
B / MHz	102.9	90.8	100.4	93.6	55.9
C / MHz	67.0	65.6	67.2	66.3	53.5
[b]$\|\mu_a\|$ / D	0.9	1.9	1.3	2.2	1.7
$\|\mu_b\|$ / D	0.4	0.6	0.5	0.3	1.4
$\|\mu_c\|$ / D	0.0	0.7	0.2	0.1	1.9
[c]$\Delta E_{CCSD(T)}$ / kJmol^{-1}	0.00	0.17	0.81	0.96	1.07
[d]ΔE_e / kJ mol^{-1}	0.03	0.00	0.86	1.39	2.94
[e]ΔE_0 / kJ mol^{-1}	0.15	0.03	0.00	1.00	2.40
[f]E_B / kJ mol^{-1}	-55.2	-55.2	-54.5	-55.9	-53.8
Isomer	VI	VII	VIII	IX	X
	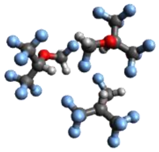	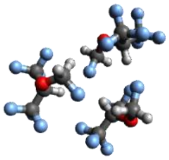	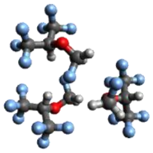	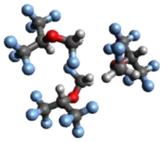	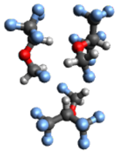
A / MHz	112.0	129.3	121.9	118.0	138.4
B / MHz	89.9	81.1	78.1	80.6	71.6
C / MHz	59.5	63.3	57.3	57.7	59.7
$\|\mu_a\|$ / D	1.1	1.2	4.9	4.9	0.2
$\|\mu_b\|$ / D	0.2	0.2	0.8	0.9	3.5
$\|\mu_c\|$ / D	1.9	0.8	1.5	1.6	0.2
$\Delta E_{CCSD(T)}$ / kJmol^{-1}	1.22	1.72	2.14	2.44	2.51
ΔE_e / kJ mol^{-1}	1.08	1.31	2.42	2.66	3.15
ΔE_0 / kJ mol^{-1}	1.19	1.07	1.74	1.97	2.77
E_B / kJ mol^{-1}	-54.2	-53.1	-52.7	-52.8	-53.3

[a]Rotational constants. [b]Magnitude of the dipole moment components. [c]CCSD(T) energy difference calculated at the B3LYP-D3(BJ)/def2-TZVP level of theory. [d]Electronic energy difference for the calculated equilibrium structures. [e]Zero-point corrected energy at B3LYP-D3(BJ)/def2-TZVP level of theory. [f]BSSE binding energies (corrected) at the same level of theory.

Table S2. Error percentage in the rotational constants for the sevoflurane dimers and trimer.

	HOMODIMER		HETERODIMER		TRIMER			
		%		%	**II**	**%**	**IV**	**%**
B3LYP-D3(BJ)/def2-TZVP	304.53	1.06	300.89	1.25	115.41	**0.24**	113.03	**2.30**
	170.05	1.20	174.34	0.70	90.82	**1.17**	93.60	**1.85**
	166.21	1.32	165.47	1.07	65.61	**0.89**	66.31	**0.17**
B3LYP-D3/6-311++g(d,p)	304.78	0.98	301.15	1.17				
	176.89	2.77	179.12	2.03				
	173.07	2.75	171.4	2.48				
EXP	307.789308[1]		304.70027		115.6893			
	172.119904		175.56391		91.89654			
	168.437022		167.25459		66.20059			

[1] Seifert, N. A.; Pérez, C.; Neill, J. L.; Pate, B. H.; Vallejo-López, M.; Lesarri, A.; Cocinero, E. J.; Castaño, F. Chiral Recognition and Atropisomerism in the Sevoflurane Dimer. *Phys. Chem. Chem. Phys.* **2015**, *17* (28), 18282–18287. https://doi.org/10.1039/C5CP01025J.

Table S3. Cartesian coordinates for the observed sevoflurane trimer (isomer II) at the B3LYP-D3(BJ)/def2-TZVP level of theory.

C	-3.09	-1.80	1.13
F	-3.06	-1.11	2.29
F	-1.99	-2.58	1.11
F	-4.15	-2.60	1.14
C	-3.07	-0.85	-0.07
O	-3.19	-1.62	-1.24
C	-2.18	-1.48	-2.17
F	-1.05	-2.20	-1.77
H	-1.86	-0.44	-2.30
H	-2.54	-1.93	-3.10
H	-2.12	-0.30	-0.04
C	-4.19	0.21	-0.05
F	-3.98	1.10	0.94
F	-4.19	0.88	-1.21
F	-5.40	-0.33	0.12
F	-0.22	-0.08	1.30
C	0.73	-0.88	1.94
O	1.80	-1.10	1.09
C	1.60	-2.05	0.06
H	0.55	-2.26	-0.12
C	2.15	-1.42	-1.23
F	2.06	-2.28	-2.26
F	1.41	-0.34	-1.54
F	3.42	-1.03	-1.11
C	2.28	-3.37	0.46
F	3.60	-3.24	0.62
F	2.06	-4.33	-0.45
F	1.76	-3.79	1.63
H	1.11	-0.32	2.79
H	0.22	-1.80	2.23
F	0.91	2.65	2.37
C	1.84	2.88	1.42
F	2.27	4.14	1.58
F	2.87	2.05	1.67
C	1.23	2.62	0.04
O	0.07	3.40	-0.13
C	-1.14	2.75	0.07
F	-1.34	1.80	-0.93
H	-1.92	3.50	-0.02
H	-1.18	2.22	1.02
H	1.02	1.55	-0.03
C	2.17	2.96	-1.13
F	2.44	4.26	-1.20
F	3.32	2.29	-1.03
F	1.58	2.60	-2.29

Table S4. Observed rotational transitions and residuals (in MHz) for Sevoflurane trimer II.

J'	K_a'	K_c'	J''	K_a''	K_c''	Obs.	Obs.−Cal.
26	0	26	25	0	25	3478.0251	−0.0003
26	1	26	25	1	25	3478.0251	−0.0003
20	10	10	19	10	9	3473.3361	0.0056
25	2	23	24	2	22	3488.3371	0.0013
25	3	23	24	3	22	3488.3371	0.0013
24	4	20	23	4	19	3499.0906	0.0002
24	5	20	23	5	19	3499.0906	0.0002
25	1	24	24	1	23	3416.9620	−0.0003
25	2	24	24	2	23	3416.9620	−0.0003
24	3	21	23	3	20	3427.4306	0.0007
24	4	21	23	4	20	3427.4306	0.0007
23	4	19	22	4	18	3366.7692	−0.0022
23	5	19	22	5	18	3366.7692	−0.0022
24	2	22	23	2	21	3355.9519	0.0017
24	3	22	23	3	21	3355.9519	0.0017
20	12	9	19	12	8	3343.4483	0.0015
25	0	25	24	0	24	3345.6326	0.0009
25	1	25	24	1	24	3345.6326	0.0009
20	11	10	19	11	9	3350.1306	0.0007
20	13	7	19	13	6	3326.9095	−0.0220
20	13	8	19	13	7	3324.3942	0.0014
20	10	11	19	10	10	3324.2667	0.0049
21	8	14	20	8	13	3322.4687	0.0071
22	2	20	21	2	19	3091.1797	−0.0018
22	3	20	21	3	19	3091.1797	−0.0018
21	4	17	20	4	16	3102.1824	0.0022
21	5	17	20	5	16	3102.1824	0.0023
23	1	23	22	1	22	3080.8434	0.0006
23	0	23	22	0	22	3080.8434	0.0006
18	8	10	17	8	9	3054.3294	−0.0038
21	3	18	20	3	17	3030.3539	0.0029
21	4	18	20	4	17	3030.3539	0.0029
20	5	15	19	5	14	3042.4342	−0.0153
20	6	15	19	6	14	3042.4342	−0.0056
19	6	13	18	6	12	2985.0666	0.0053
19	7	13	18	7	12	2984.4690	−0.0036
18	12	6	17	12	5	2988.0618	−0.0031
18	9	10	17	9	9	2984.2249	−0.0001
20	4	16	19	4	15	2969.9181	−0.0002
20	5	16	19	5	15	2969.9181	0.0001
18	13	6	17	13	5	2970.1883	−0.0276
18	14	4	17	14	3	2957.9742	0.0060
18	14	5	17	14	4	2957.9742	0.0136
21	2	19	20	2	18	2958.7980	−0.0010
21	3	19	20	3	18	2958.7980	−0.0010

26	6	20	25	6	19	3907.7888	−0.0052
26	7	20	25	7	19	3907.7888	−0.0052
25	9	17	24	9	16	3923.0055	0.0028
25	8	17	24	8	16	3923.0750	−0.0021
24	21	3	23	21	2	3923.2114	0.0013
24	21	4	23	21	3	3923.2114	0.0013
24	20	4	23	20	3	3930.9738	0.0020
24	20	5	23	20	4	3930.9738	0.0020
16	1	15	15	1	14	2225.4316	0.0000
16	2	15	15	2	14	2225.4316	0.0000
13	6	7	12	6	6	2233.6451	−0.0008
15	3	12	14	3	11	2236.5413	−0.0052
15	4	12	14	4	11	2236.5413	0.0123
14	6	9	13	6	8	2248.5266	0.0027
14	5	9	13	5	8	2261.6668	0.0006
17	0	17	16	0	16	2286.4639	−0.0014
17	1	17	16	1	16	2286.4639	−0.0014
16	2	14	15	2	13	2296.9318	−0.0017
16	3	14	15	3	13	2296.9318	−0.0016
14	7	8	13	7	7	2305.4571	−0.0059
15	4	11	14	4	10	2309.6604	−0.0099
14	9	6	13	9	5	2325.6614	−0.0024
17	1	16	16	1	15	2357.8216	−0.0009
17	2	16	16	2	15	2357.8216	−0.0009
16	3	13	15	3	12	2368.7704	−0.0040
16	4	13	15	4	12	2368.7704	0.0002
18	0	18	17	0	17	2418.8606	−0.0019
18	1	18	17	1	17	2418.8606	−0.0019
14	7	7	13	7	6	2425.5039	0.0012
17	2	15	16	2	14	2429.2950	−0.0014
17	3	15	16	3	14	2429.2950	−0.0014
16	5	12	15	5	11	2441.3084	−0.0023
16	4	12	15	4	11	2441.4150	0.0024
29	4	25	28	4	24	4160.8126	0.0011
29	5	25	28	5	24	4160.8126	0.0011
24	12	12	23	12	11	4172.5697	−0.0020
28	6	22	27	6	21	4172.1958	0.0004
28	7	22	27	7	21	4172.1958	0.0004
27	8	19	26	8	18	4186.0888	−0.0050
27	9	19	26	9	18	4186.0888	0.0008
28	7	21	27	7	20	4244.6650	−0.0021
28	8	21	27	8	20	4244.6650	−0.0020
29	6	23	28	6	22	4304.4389	0.0025
29	7	23	28	7	22	4304.4389	0.0025
26	11	15	25	11	14	4305.6067	−0.0011
28	8	20	27	8	19	4317.8500	−0.0049
28	9	20	27	9	19	4317.8500	−0.0033
27	10	17	26	10	16	4338.8345	0.0007

25	13	12	24	13	11	4342.4965	−0.0210
26	13	14	25	13	13	4346.3438	0.0018
30	5	25	29	5	24	4364.8121	−0.0068
30	6	25	29	6	24	4364.8121	−0.0068
26	15	12	25	15	11	4366.2611	0.0091
17	3	14	16	3	13	2501.0448	−0.0023
17	4	14	16	4	13	2501.0448	−0.0013
15	9	7	14	9	6	2499.8875	0.0006
18	1	17	17	1	16	2490.2125	−0.0018
18	2	17	17	2	16	2490.2125	−0.0018
16	6	11	15	6	10	2514.8841	−0.0001
16	5	11	15	5	10	2516.4555	−0.0084
19	0	19	18	0	18	2551.2592	−0.0002
19	1	19	18	1	18	2551.2592	−0.0002
18	2	16	17	2	15	2561.6656	−0.0004
18	3	16	17	3	15	2561.6656	−0.0004
15	7	8	14	7	7	2580.8654	0.0020
16	7	10	15	7	9	2586.9794	0.0015
15	8	7	14	8	6	2592.2062	−0.0035
19	1	18	18	1	17	2622.6055	−0.0011
19	2	18	18	2	17	2622.6055	−0.0011
18	3	15	17	3	14	2633.3497	0.0013
18	4	15	17	4	14	2633.3497	0.0015
15	0	15	14	0	14	2021.6618	−0.0084
15	1	15	14	1	14	2021.6618	−0.0084
14	2	12	13	2	11	2032.2375	−0.0037
14	3	12	13	3	11	2032.2375	−0.0013
12	5	7	11	5	6	2020.8373	−0.0013
13	5	9	12	5	8	2044.6576	0.0191
15	1	14	14	1	13	2093.0384	−0.0036
15	2	14	14	2	13	2093.0384	−0.0036
14	4	11	13	4	10	2104.3256	−0.0026
14	3	11	13	3	10	2104.4018	0.0033
13	5	8	12	5	7	2141.0816	0.0100
16	0	16	15	0	15	2154.0663	−0.0016
16	1	16	15	1	15	2154.0663	−0.0016
14	5	10	13	5	9	2177.2405	0.0249
14	4	10	13	4	9	2178.5162	0.0288
13	8	6	12	8	5	2161.1282	0.0020
15	2	13	14	2	12	2164.5764	−0.0038
15	3	13	14	3	12	2164.5764	−0.0033
15	6	9	14	6	8	2482.9815	−0.0020
16	8	9	15	8	8	2644.5947	−0.0068
17	6	12	16	6	11	2646.7447	−0.0008
16	11	6	15	11	5	2648.4742	−0.0067
16	11	5	15	11	4	2649.9991	0.0087
16	10	7	15	10	6	2664.2149	−0.0148
16	9	8	15	9	7	2669.6244	0.0023

20	0	20	19	0	19	2683.6567	0.0009
20	1	20	19	1	19	2683.6567	0.0009
19	2	17	18	2	16	2694.0394	−0.0011
19	3	17	18	3	16	2694.0394	−0.0011
18	4	14	17	4	13	2705.5336	0.0250
18	5	14	17	5	13	2705.5336	0.0317
16	7	9	15	7	8	2709.7591	0.0103
17	7	11	16	7	10	2720.7501	−0.0072
17	6	11	16	6	10	2726.3364	0.0044
16	9	7	15	9	6	2738.0952	−0.0076
20	1	19	19	1	18	2754.9986	−0.0007
20	2	19	19	2	18	2754.9986	−0.0007
19	3	16	18	3	15	2765.6706	0.0010
19	4	16	18	4	15	2765.6706	0.0011
16	8	8	15	8	7	2774.5826	−0.0007
18	5	13	17	5	12	2778.6902	−0.0021
17	14	3	16	14	2	2786.1194	−0.0035
17	14	4	16	14	3	2786.1194	−0.0024
17	8	10	16	8	9	2788.4827	0.0035
21	0	21	20	0	20	2816.0469	−0.0050
21	1	21	20	1	20	2816.0469	−0.0050
17	7	10	16	7	9	2825.0616	−0.0118
17	11	7	16	11	6	2825.6971	−0.0055
20	2	18	19	2	17	2826.4146	−0.0038
20	3	18	19	3	17	2826.4146	−0.0038
17	9	9	16	9	8	2831.6361	0.0022
19	4	15	18	4	14	2837.6896	−0.0002
19	5	15	18	5	14	2837.6896	0.0014
17	10	8	16	10	7	2839.3735	0.0003
18	7	12	17	7	11	2852.8680	0.0000
18	6	12	17	6	11	2854.7438	−0.0066
17	10	7	16	10	6	2876.9257	−0.0032
20	3	17	19	3	16	2898.0041	−0.0009
20	4	17	19	4	16	2898.0041	−0.0009
18	8	11	17	8	10	2925.8131	0.0005
17	8	9	16	8	8	2928.1764	0.0012
18	16	2	17	16	1	2940.8441	0.0013
18	16	3	17	16	2	2940.8441	0.0013
17	9	8	16	9	7	2941.5670	−0.0019
18	7	11	17	7	10	2942.2024	−0.0012
18	11	8	17	11	7	3003.5242	−0.0005
18	10	9	17	10	8	3009.6225	0.0035
18	11	7	17	11	6	3020.6715	−0.0028
18	10	8	17	10	7	3086.1433	0.0034
20	7	14	19	7	13	3116.0472	−0.0059
20	6	14	19	6	13	3116.2272	0.0006
19	15	4	18	15	3	3119.9194	0.0026
19	15	5	18	15	4	3119.9194	0.0046

23	2	22	22	2	21	3152.1882	0.0105
23	1	22	22	1	21	3152.1882	0.0105
22	3	19	21	3	18	3162.7023	−0.0027
22	4	19	21	4	18	3162.7023	−0.0027
19	12	8	18	12	7	3164.5854	0.0119
19	8	11	18	8	10	3167.3048	0.0017
19	10	10	18	10	9	3171.7842	0.0051
21	5	16	20	5	15	3174.5158	−0.0007
21	6	16	20	6	15	3174.5158	0.0018
19	11	9	18	11	8	3179.4728	−0.0013
20	8	13	19	8	12	3191.1455	0.0048
24	1	24	23	1	23	3213.2377	0.0002
24	0	24	23	0	23	3213.2377	0.0002
19	11	8	18	11	7	3222.4724	−0.0017
23	2	21	22	2	20	3223.5647	−0.0007
23	3	21	22	3	20	3223.5647	−0.0007
22	4	18	21	4	17	3234.4663	−0.0003
22	5	18	21	5	17	3234.4663	−0.0002
20	9	12	19	9	11	3264.9832	0.0013
19	9	10	18	9	9	3275.5390	−0.0010
20	16	4	19	16	3	3281.9839	0.0031
20	16	5	19	16	4	3281.9839	0.0036
20	8	12	19	8	11	3282.8002	−0.0080
24	1	23	23	1	22	3284.5705	0.0004
24	2	23	23	2	22	3284.5705	0.0004
19	10	9	18	10	8	3291.3373	0.0021
23	3	20	22	3	19	3295.0651	0.0000
23	4	20	22	4	19	3295.0651	0.0000
22	5	17	21	5	16	3306.6540	0.0014
22	6	17	21	6	16	3306.6540	0.0020
20	12	8	19	12	7	3363.6168	0.0032
22	6	16	21	6	15	3379.5903	−0.0055
22	7	16	21	7	15	3379.5903	0.0077
21	16	5	20	16	4	3454.4786	−0.0016
21	16	6	20	16	5	3454.4786	0.0018
22	7	15	21	7	14	3454.0041	0.0026
22	8	15	21	8	14	3453.7895	0.0000
21	9	12	20	9	11	3509.6449	0.0024
23	6	17	22	6	16	3511.5272	−0.0010
23	7	17	22	7	16	3511.5272	0.0024
21	11	11	20	11	10	3512.3496	−0.0025
21	12	10	20	12	9	3520.1121	0.0074
22	9	14	21	9	13	3529.6865	0.0093
22	8	14	21	8	13	3532.1369	0.0008
22	10	13	21	10	12	3604.4450	−0.0021
27	0	27	26	0	26	3610.4203	0.0018
27	1	27	26	1	26	3610.4203	0.0018
26	2	24	25	2	23	3620.7194	−0.0024

26	3	24	25	3	23	3620.7194	−0.0024
21	10	11	20	10	10	3622.9139	−0.0113
22	9	13	21	9	12	3623.5843	−0.0090
22	16	6	21	16	5	3628.4794	−0.0051
22	16	7	21	16	6	3628.4794	0.0139
25	4	21	24	4	20	3631.4229	0.0024
25	5	21	24	5	20	3631.4229	0.0024
24	6	18	23	6	17	3643.5529	−0.0014
24	7	18	23	7	17	3643.5529	−0.0006
22	15	8	21	15	7	3643.9061	0.0029
22	15	7	21	15	6	3644.2113	0.0212
23	9	15	22	9	14	3660.6992	0.0056
23	8	15	22	8	14	3661.4899	−0.0120
22	14	9	21	14	8	3663.3492	−0.0028
22	11	12	21	11	11	3664.6595	0.0038
22	14	8	21	14	7	3666.5030	−0.0002
27	2	26	26	2	25	3681.7459	0.0004
27	1	26	26	1	25	3681.7459	0.0004
22	13	10	21	13	9	3683.9159	−0.0001
22	12	11	21	12	10	3691.0836	−0.0009
26	3	23	25	3	22	3692.1675	−0.0021
26	4	23	25	4	22	3692.1675	−0.0021
25	5	20	24	5	19	3703.3092	−0.0029
25	6	20	24	6	19	3703.3092	−0.0029
23	10	14	22	10	13	3737.2908	−0.0114
28	0	28	27	0	27	3742.8104	−0.0006
28	1	28	27	1	27	3742.8104	−0.0006
23	9	14	22	9	13	3744.8851	−0.0006
27	2	25	26	2	24	3753.1071	−0.0010
27	3	25	26	3	24	3753.1071	−0.0010
26	4	22	25	4	21	3763.7567	−0.0026
26	5	22	25	5	21	3763.7567	−0.0026
25	6	19	24	6	18	3775.6445	−0.0040
25	7	19	24	7	18	3775.6445	−0.0038
23	18	5	22	18	4	3778.1373	−0.0161
23	18	6	22	18	5	3778.1373	−0.0159
22	12	10	21	12	9	3783.9356	0.0003
23	17	6	22	17	5	3789.7332	0.0003
23	17	7	22	17	6	3789.7332	0.0057
24	9	16	23	9	15	3791.7539	0.0032
24	8	16	23	8	15	3791.9986	−0.0032
28	1	27	27	1	26	3814.1338	−0.0027
28	2	27	27	2	26	3814.1338	−0.0027
23	15	9	22	15	8	3822.4002	0.0025
22	11	11	21	11	10	3822.9114	0.0043
27	3	24	26	3	23	3824.5423	−0.0007
27	4	24	26	4	23	3824.5423	−0.0007
26	5	21	25	5	20	3835.5819	−0.0032

26	6	21	25	6	20	3835.5819	−0.0032
23	14	10	22	14	9	3844.1546	0.0032
25	7	18	24	7	17	3848.7239	0.0015
25	8	18	24	8	17	3848.7239	0.0061
23	10	13	22	10	12	3852.0636	−0.0042
24	10	15	23	10	14	3868.4723	0.0175
29	1	29	28	1	28	3875.2058	0.0029
29	0	29	28	0	28	3875.2058	0.0029
28	2	26	27	2	25	3885.4942	−0.0002
28	3	26	27	3	25	3885.4942	−0.0002
28	3	25	27	3	24	3956.9192	0.0011
28	4	25	27	4	24	3956.9192	0.0011
27	5	22	26	5	21	3967.8797	0.0043
27	6	22	26	6	21	3967.8797	0.0043
23	11	12	22	11	11	3970.3080	0.0037
23	12	11	22	12	10	3991.8564	0.0009
30	1	30	29	1	29	4007.5943	0.0001
30	0	30	29	0	29	4007.5943	0.0001
29	2	27	28	2	26	4017.8796	−0.0011
29	3	27	28	3	26	4017.8796	−0.0011
28	4	24	27	4	23	4028.4548	−0.0012
28	5	24	27	5	23	4028.4548	−0.0012
27	6	21	26	6	20	4039.9776	−0.0015
27	7	21	26	7	20	4039.9776	−0.0015
25	11	15	24	11	14	4076.6618	0.0013
25	10	15	24	10	14	4084.8317	0.0052
28	5	23	27	5	22	4100.1880	0.0085
28	6	23	27	6	22	4100.1880	0.0085
24	13	11	23	13	10	4133.5691	−0.0015
30	3	28	29	3	27	4150.2686	0.0017
30	2	28	29	2	27	4150.2686	0.0017
25	11	14	24	11	13	4194.5568	−0.0029
25	13	13	24	13	12	4194.5568	−0.0076
30	3	27	29	3	26	4221.6736	0.0018
30	4	27	29	4	26	4221.6736	0.0018
30	4	26	29	4	25	4293.1697	−0.0009
30	5	26	29	5	25	4293.1697	−0.0009
26	14	13	25	14	12	4374.1188	0.0069
29	7	22	28	7	21	4376.7803	0.0052
29	8	22	28	8	21	4376.7803	0.0052
27	12	16	26	12	15	4416.2313	−0.0024
27	11	16	26	11	15	4424.9200	−0.0204
27	22	5	26	22	4	4426.5991	0.0130
27	22	6	26	22	5	4426.5991	0.0130
30	6	24	29	6	23	4436.6984	0.0011
30	7	24	29	7	23	4436.6984	0.0011
29	8	21	28	8	20	4449.7235	−0.0019
29	9	21	28	9	20	4449.7235	−0.0015

27	18	10	26	18	9	4479.2604	−0.0215
26	14	12	25	14	11	4483.6366	0.0042
27	13	15	26	13	14	4488.7382	0.0048
27	17	11	26	17	10	4500.7996	0.0097
27	17	10	26	17	9	4502.4988	0.0093
30	7	23	29	7	22	4508.9244	0.0000
30	8	23	29	8	22	4508.9244	0.0000
29	9	20	28	9	19	4523.6343	−0.0018
29	10	20	28	10	19	4523.6343	0.0054
28	12	17	27	12	16	4546.6568	0.0045
28	11	17	27	11	16	4549.8594	−0.0208
30	8	22	29	8	21	4581.6788	−0.0011
30	9	22	29	9	21	4581.6788	−0.0010
28	23	5	27	23	4	4588.8229	−0.0149
28	23	6	27	23	5	4588.8229	−0.0149
28	20	8	27	20	7	4622.8067	0.0087
28	20	9	27	20	8	4622.8067	0.0120
29	12	18	28	12	17	4676.6738	0.0032
29	11	18	28	11	17	4677.7847	−0.0057
20	12	9	19	10	10	4680.5964	−0.0053
29	13	17	28	13	16	4756.0027	0.0005
29	12	17	28	12	16	4765.2129	0.0037
28	13	15	27	13	14	4777.4241	−0.0049
29	17	13	28	17	12	4867.1526	−0.0198
28	14	14	27	14	13	4872.0593	0.0065
29	15	15	28	15	14	4877.9430	0.0123
29	13	16	28	13	15	4879.7033	0.0198
